# Geostatistical modeling of malaria prevalence among under-five children in Rwanda

**DOI:** 10.1186/s12889-021-10305-x

**Published:** 2021-02-17

**Authors:** Jean Damascene Nzabakiriraho, Ezra Gayawan

**Affiliations:** 1 African Institute for Mathematical Sciences, Kigali, Rwanda; 2 Department of Statistics, Federal University of Technology, Akure, Nigeria

**Keywords:** Geostatistical modeling, Exceedance probability, Malaria prevalence, Rwanda, Demographic and health survey

## Abstract

**Background:**

Malaria has continued to be a life-threatening disease among under-five children in sub-Saharan Africa. Recent data indicate rising cases in Rwanda after some years of decline. We aimed at estimating the spatial variations in malaria prevalence at a continuous spatial scale and to quantify locations where the prevalence exceeds the thresholds of 5% and 10% across the country. We also consider the effects of some socioeconomic and climate variables.

**Methods:**

Using data from the 2014-2015 Rwanda Demographic and Health Survey, a geostatistical modeling technique based on stochastic partial differential equation approach was used to analyze the geospatial prevalence of malaria among under-five children in Rwanda. Bayesian inference was based on integrated nested Laplace approximation.

**Results:**

The results demonstrate the uneven spatial variation of malaria prevalence with some districts including Kayonza and Kirehe from Eastern province; Huye and Nyanza from Southern province; and Nyamasheke and Rusizi from Western province having higher chances of recording prevalence exceeding 5%. Malaria prevalence was found to increase with rising temperature but decreases with increasing volume for rainfall. The findings also revealed a significant association between malaria and demographic factors including place of residence, mother’s educational level, and child’s age and sex.

**Conclusions:**

Potential intervention programs that focus on individuals living in rural areas, lowest wealth quintile, and the locations with high risks should be reinforced. Variations in climatic factors particularly temperature and rainfall should be taken into account when formulating malaria intervention programs in Rwanda.

## Background

Malaria, which is endemic in both tropics and subtropics, remained one of the preventable and curable diseases that has continued to threaten human health and survival especially among young children [1]. It is one of the world’s most deadliest diseases that causes significant public health challenge. In 2018, nearly 50% of the world’s population was at risk of malaria but the majority of the recorded cases and deaths happened in sub-Saharan Africa, where the majority of the victims were children below the age of five years [2, 3]. Malaria does not only put pressure on health care systems, but also has direct consequence on individuals and households because of the loss of income and other economic aftermath [4, 5]. There has been several efforts for over a century, to control malaria incidence especially in endemic regions but the prevalence and fatality rates have not been significantly reduced in sub-Saharan Africa. The total funding for malaria control and elimination, including the contributions of governments of endemic countries reached an estimated USD 2.7 billion in 2018 [3]. However, malaria is still among the main public health problems in least developed regions of the world [6]. Of the estimated 300 million acute cases each year, between 1.1 and 2.7 million end in death. In 2013, there were about 198 million cases of which an estimated 584,000 resulted in death, with 90% of these deaths occurring in Africa [7]. In 2017, fatalities among young children under age 5 years was about 61% of the total deaths, translating to a daily toll of nearly 730 deaths of these children with the majority occurring in sub-Saharan Africa [8].

In Rwanda, malaria has, for a long time, remained a major cause of morbidity and mortality, with regular occurrence in high-altitude areas. Most of the cases occur around May-June and November-December, and studies have shown that it is related to altitude because of the high prevalence in lowlands than in highlands areas [9]. Aside favorable climate, factors such as proximity to marshlands, irrigation schemes, and cross border movement of people impact on the transmission especially in the Southern and Eastern parts of the country [10, 11]. In the past decade, there has been a drastic reduction in the burden of malaria throughout Rwanda [12]. This is evident from the recorded 86% decline in incidence between 2005 and 2011; 87% decline in hospital outpatient due to malaria between 2005 and 2011; and 74% decline in inpatient deaths from malaria over the same period. The number of positive test also reduced by 71% [12]. Further, among under-five children, malaria prevalence decreased from 2.6% to 1.4% between 2008 and 2010 while among pregnant women, it reduced from 1.4% to 0.7% over the same period [13]. However, since 2012, there has been an upturn of the recorded gain as the country has experienced a surge in malaria cases reaching about 1,597,143 cases in 2013 from 962,000 reported in 2012 [14]. Malaria morbidity rate was 5.8% in 2012 but increased to 18.3% in 2015 [14], and deaths of under-five children resulted from malaria have been increasing [15]. Some authors have attributed the sudden increase to climatic change, environmental modification, human behavior, and insecticide resistance [16]. However, the efficacy antimalarial drugs has caused trend in mortality due to malaria among the populace to remain at about 5% for some years [14].

Several studies have evaluated malaria prevalence and the associated socio-demographic risk factors among under-five children in Rwanda [17–22]. For instance, households with multiple occupants were identified to have higher risk of malaria but the risks were lower for those whose heads were educated and of higher socioeconomic status [11]. Differential in malaria risk behavior especially use of bed net, and parasite density were found to be the major cause of differences in the intensity of malaria transmission between two sites of different malaria transmission in the country [23]. However, only few studies have linked malaria prevalence to climatic conditions in the country. Notwithstanding that the entire population is at risk, there is heterogeneous spatial and temporal variations in mosquito presence and in malaria prevalence across the country as evident from different endemic settings [12, 24]. Hospitals in the Eastern province reported higher rates of malaria admissions and deaths than those in the other provinces [12]. Consequently, it is essential to study the pattern of spatial variations in malaria prevalence at small scale level across the country. A vital component of disease mapping is that it provides an easy-to-understand visual summary of the spatial information and identifies the geographical distributions that may be difficult to achieve if tabular representations are employed. Most of the existing studies have quantified malaria prevalence and its risk factors at larger scales whereas a spatial mapping at smaller geographic units are needed for effective and efficient interventions. Allocation of scarce resources to benefit places where they are mostly needed can be enhanced when findings from an analysis that presents local prediction of malaria prevalence across the country are taken into consideration [25, 26]. Proper understanding of the dynamics of climatic conditions and the spatial variation of malaria prevalence in Rwanda would support cost-effective use of available meagre resources towards eradication of the malaria.

To this end, malaria data extracted from the 2014-2015 RDHS are utilized to analyze the geospatial prevalence of malaria among under-five children in Rwanda. We compute and map the exceedance probabilities at 5% and 10% thresholds in order to determine the specific locations, across the country, where malaria prevalence exceeds these thresholds. Moreover, we examined the effects of climatic, environmental, and demographic factors while analysing the spatial patterns of malaria prevalence. The geostatistical modeling technique was based on stochastic partial differential equation (SPDE), a Bayesian spatial technique that allows for continuous mapping of a spatial phenomenon across space. The model is considered appropriate because of its capability to incorporate several confounding variables of different types while quantifying the effects of spatial processes. We used the integrated nested Laplace approximation (INLA) to obtain approximations to the posterior marginals of all parameters of interest.

## Methods

### Data

**Fig. 1:**
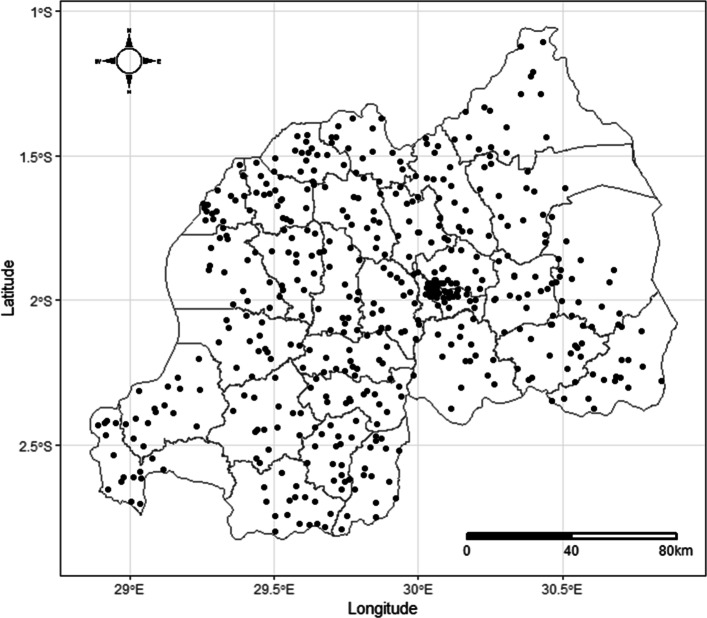
Map of Rwanda showing the distribution of selected clusters for data collection during the 2014-2015 RDHS (Source: Authors’ creation)

This study used data from the 2014-2015 RDHS conducted by the National Institute of Statistics of Rwanda (NISR) in collaboration with the Ministry of Health (MoH) and the Rwanda Biomedical Center (RBC) from November 9, 2014 to April 8, 2015. The national survey aimed at obtaining information on demographic and health indicators among women of reproductive age and their children under the age of five years. The survey was also meant to estimate the prevalence of anemia and malaria among these set of people. Like for other similar survey, a two-stage sampling design was adopted. At the first stage, clusters consisting of enumeration areas (EAs) delineated for the 2012 Rwanda Population and Housing Census were identified. A total of 492 clusters comprising 113 in urban and 379 in rural areas as represented in Fig. 1 were selected. At the second stage, a random selection was used to determine the households to include in the survey from the sampling frame such that 26 households were selected from each EA, leading to a total sample of 12,793 households. Of these, 12,717 were found to be occupied at the time of the survey and individuals from 12,699 households successfully completed the survey questionnaires, yielding a response rate of 99.9%. All women aged 15-49 years and under-five children in the selected households were eligible for individual interviews and for malaria testing respectively.

Malaria testing for the children were based on malaria diagnostic tests, including rapid diagnostic tests (RDT) and tests using thick and thin blood smears. In the case of RDTs, a drop of blood was obtained by pricking the end of the finger, and a slide with a thick and thin blood smears was prepared and stained. These samples were transmitted to the NISR for verification and then stored at the Parasitology and Entomology Laboratory for microscopic examination of malaria parasites, before referring to the National Reference Laboratory for quality control and assurance. The outcome variable for our study is a binary response variable from the RDT test result which was recorded, for each child, as either 1 (positive) or 0 (negative). The information collected and explored for the purpose of this study includes child’s age, child’s sex, household wealth index, mother’s educational attainment, type of place of residence, use of treated bed net, bed net ownership, and age of household head. Furthermore, climate variables including temperature and rainfall were extracted from the GPS data set of the 2014-2015 RDHS, to explore their effect on malaria prevalence. Data analysis was conducted over 3,180 under-five children for whom information on malaria was available which comprise of 787 from East; 364 from Kigali city; 443 from North; 817 from South, and 769 from West.

### Statistical analysis

The SPDE model [27] implemented in the R-INLA package [28] has been used to predict malaria prevalence among under-five children across space in Rwanda. Let *Y*_*i*_ denotes a binary outcome indicating the malaria variable of *n* sampled population and let *p* be the true probability that a given child suffered from malaria. Consequently, *Y*_*i*_ is assumed to have a binomial probability distribution denoted as

1 $$  Y_{i}|p\sim\mathcal{B}(n,p).  $$

Assuming a set of covariates that contains *m* categorical and *v* continuous covariates are available, then a logit link function can be utilized to relate the covariates to the malaria data at location *s*_*i*_, expressed as follows:

2 $$ \begin{aligned} logit~(p)&=\beta_{0}+\beta_{1}m_{1}+\cdots+\beta_{m}m_{m}+f_{1}(v_{1})+\cdots\\&\quad+f_{v}(v_{v})+f_{loc}(s_{i}) \end{aligned}  $$

where *β*_0_ represents the model intercept, *β*_*m*_=(*β*_1_,⋯,*β*_*m*_) denotes the vector of linear fixed effects for the categorical covariates, *f*_*g*_,*g*=1,⋯,*v* are smooth functions assumed for the nonlinear effects of continuous covariates, and *f*_*loc*_(*s*_*i*_) denotes a spatial random effect.

To implement the spatial model and predict the variable of interest at unsampled locations, the SPDE model assumes that the values of the spatially continuous variable collected at specific locations can be approximated by utilizing a Gaussian random field (GRF). The solution of the continuous domain can thus be considered as GRF with a Matérn covariance matrix:

3 $$  (\kappa^{2}-\Delta)^{\frac{\alpha}{2}} \left(\tau x(s)\right)=\omega(s),  $$

where *x*(*s*) is a GRF and *ω*(*s*) is a Gaussian spatial white noise process. The parameters *τ* and *α* respectively control the variance and the smoothness of the GRF, while *κ*>0 is a scale parameter and *Δ* is the Laplacian defined as $\sum _{i=1}^{d}\frac {\partial ^{2}}{\partial x_{i}^{2}}$, with *d* being the dimension of the spatial domain $\mathscr {D}$. A finite element method, that divides the total spatial area into a set of non-overlapping triangles that forms a triangulated mesh of *n* nodes and *n* basis functions can be used to approximate the solution of the model. To ease computation, the Gaussian field *x* may be rewritten as a discretely indexed GMRF using a basis function defined on the triangulated mesh as follows:

4 $$ x(s)=\sum_{\kappa=1}^{\varkappa}\Psi_{\kappa}(s)x_{\kappa},  $$

where, *Ψ*_*κ*_(·) is defined as a piecewise linear function assumed to be 1 at vertex *κ* and 0 if otherwise, ϰ represent the number of vertices of the triangulated mesh and *x*_*κ*_ is the Gaussian distributed weights centralized at zero with a distribution defined as

5 $$ x=(x_{1},\cdots,x_{n})\sim \mathcal{N}\left(0,Q^{-1}\left(\tau,\kappa\right)\right).  $$

The basis functions transform the approximated *x*(*s*) from the mesh nodes to the other spatial locations of interest. The exceedance probability, the probability that malaria prevalence in a given area is greater than a defined threshold value *θ* can be expressed as *P*(*ϕ*>*θ*). In this study, two thresholds, 5% and 10% were chosen in order to estimate the cluster areas with consistent high malaria burden in Rwanda. The functions *f*_*g*_ for the nonlinear effects of the continuous covariates were modelled assuming a second-order random walk prior while the linear terms were assigned Gaussian priors with mean equal to 0 and precision equal to 0.001. Bayesian inference was through INLA as proposed by [29]. More details about the SPDE model and its implementation have been described in [25].

## Results

Table 1 presents the descriptive statistics of malaria prevalence based on respondents’ characteristics. Overall, 7.4% (235/3,180) of the children tested positive for malaria. Malaria prevalence was higher among children from rural areas when compared with their counterparts from urban areas. Out of 2,515 children from rural areas, 218 (8.7%) tested positive for malaria compared with only 17 (2.6%) children from the urban settings. Malaria prevalence was higher (9.6%) among children whose mothers had no education while there was no positive case reported among those children whose mothers attained higher education. Malaria prevalence was higher among children from the Eastern and Southern provinces of the country. Out of 787 surveyed children from the Eastern province, 118 (14.9%) tested positive while among the 817 children from the Southern province, 92 (11.3%) were positive for malaria. However, no positive case of malaria was reported among the surveyed children from the Northern province. Furthermore, the prevalence of malaria appears to be higher among older children. The prevalence was 10.4% among children aged 24-35 months and 9.6% among those 48-59 months but of the 377 children less than 12 months of age, only 17 (4.6%) tested positive.

**Table 1:** Demographic characteristics of respondents, source: RDHS 2014-2015

Parameter	Negative (%)	Positive (%)	Total
Place of residence			
Rural	2,297 (91.3)	218 (8.7)	2,515
Urban	648 (97.4)	17 (2.6)	665
Mother’s education			
No education	426 (90.4)	45 (9.6)	471
Primary	2,116 (92.3)	177 (7.7)	2,293
Secondary	326 (96.2)	13 (3.8)	339
Higher	77 (100.0)	0 (0.0)	77
Region			
East	669 (85.1)	118 (14.9)	787
Kigali city	359 (98.6)	5 (1.4)	364
North	443 (100.0)	0(0.0)	443
South	725 (88.7)	92 (11.3)	817
West	749 (97.4)	20 (2.6)	769
Child’age group			
<12 months	377 (95.4)	18(4.6)	395
12-23 months	699 (95.6)	32(4.4)	731
24-35 months	661 (89.6)	77(10.4)	738
36-47 month	688 (92.9)	53(7.1)	741
48-59 moths	520 (90.4)	55(9.6)	575
Wealth index			
Poorest	682 (87.7)	96 (12.3)	778
Poorer	625 (91.1)	61 (8.9)	686
Mild-range	565 (92.6)	45 (7.4)	610
Richer	499 (94.7)	28 (5.3)	527
Richest	574 (99.1)	5 (0.9)	579
Sex			
Male	1,507 (92.5)	123 (7.5)	1,630
Female	1,438 (92.8)	112 (7.2)	1,550
Bed-net ownership			
Yes	2,513 (93.0)	188 (7.0)	2,701
No	432 (90.2)	47 (9.8)	479
Use of bed-net			
No	344 (92.0)	30 (8.0)	374
All children	1,926 (93.5)	133 (6.5)	2,059
Some children	243 (90.7)	25 (9.3)	268
No net in household	432 (90.2)	47 (9.8)	479
Total	2,945 (92.6)	235 (7.4)	3,180

Children from the richest households were less affected by malaria as only 0.9% of children in this category suffered from malaria whereas, the prevalence was as high as 12.3% among those from the poorest households. The findings further reveal that the proportion of male children with malaria (7.5%) is almost the same as for female children (7.2%). Among children from households having bed nets, 7.0% tested positive for malaria compared with 9.8% of those without bed net. The proportion of children with malaria varies with the use of bed nets within households such that about 9.8% of children who did not sleep under bed nets suffered from malaria compared with 6.5% of those who slept under bed nets.

Table 2 presents results of the linear effects showing the posterior means, standard deviations, and 95% credible intervals. The findings reveal that mother’s educational level is significantly related to the likelihood of having malaria. Children whose mothers attained at least primary educational level are less likely to have malaria when compared with those whose mothers have no education. Children from urban settings are less likely to have malaria when compared with those from rural areas. Similarly, the findings show that male children are less likely to have malaria when compared with the female children. Children from at least poorer households are less likely, compared with those from the poorest households, to have malaria. Findings on bed net reveal that ownership and use of bet net reduces the chances of contracting malaria such that children from households where all the children slept under bet net in the night before the survey were less likely, but the estimate for households where some of the children slept under bed net is not significant.

**Table 2:** Estimated posterior means with 95% credible interval for the linear effects

Parameter	mean	sd	0.025quant	0.975quant
Place of residence				
Rural	1			
Urban	-0.108	0.048	-0.203	-0.014
Mother’s education				
No education	1			
Primary	-0.152	0.029	-0.208	-0.096
Secondary/higher	-0.149	0.058	-0.264	-0.035
Household wealth index				
Poorest	1			
Poorer	-0.143	0.044	-0.231	-0.056
Mild-range	-0.154	0.047	-0.246	-0.062
Richer	-0.164	0.050	-0.263	-0.066
Richest	-0.163	0.053	-0.268	-0.059
Sex of child				
Female	1			
Male	-0.103	0.031	-0.164	-0.043
Bed-net ownership				
No	1			
Yes	-0.178	0.029	-0.235	-0.122
Bed-net usage				
No child	1			
All children	-0.145	0.031	-0.205	-0.085
Some children	-0.126	0.068	-0.260	0.007

**Fig. 2:**
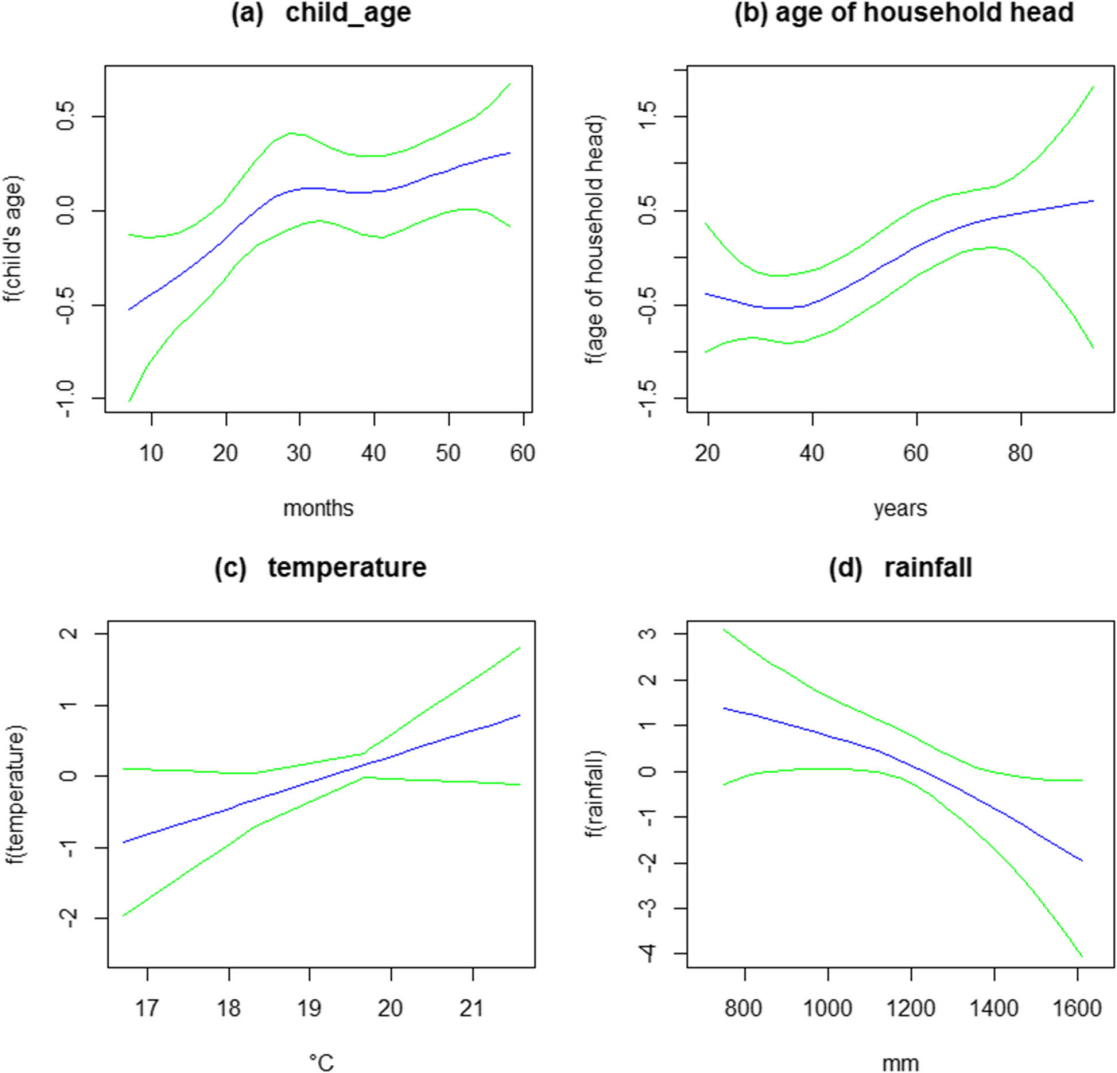
Posterior means of nonlinear effects of child’s age in months **a**, age of household head in years **b**, Temperature in degree cercius **c**, and rainfall in milimeter **d**

The results for the non-linear effects of child’s age, age of household head, temperature, and rainfall are presented in Fig. 2. The Figure presents, in each case, the posterior means and 95% credible intervals. The study findings reveal a positive association between the likelihood of having malaria and child’s age, demonstrating that as the child advances in age, the likelihood of contracting malaria increases. The likelihood of contracting malaria increases for children whose household head is more than 40 years of age. Prior to this age, the chances are lower. Regarding temperature, the findings demonstrate higher chances of having malaria with increasing temperature but an inverse relationship was evident in the case of rainfall such that children are less likely to test positive for malaria with increasing rainfall.

**Fig. 3:**
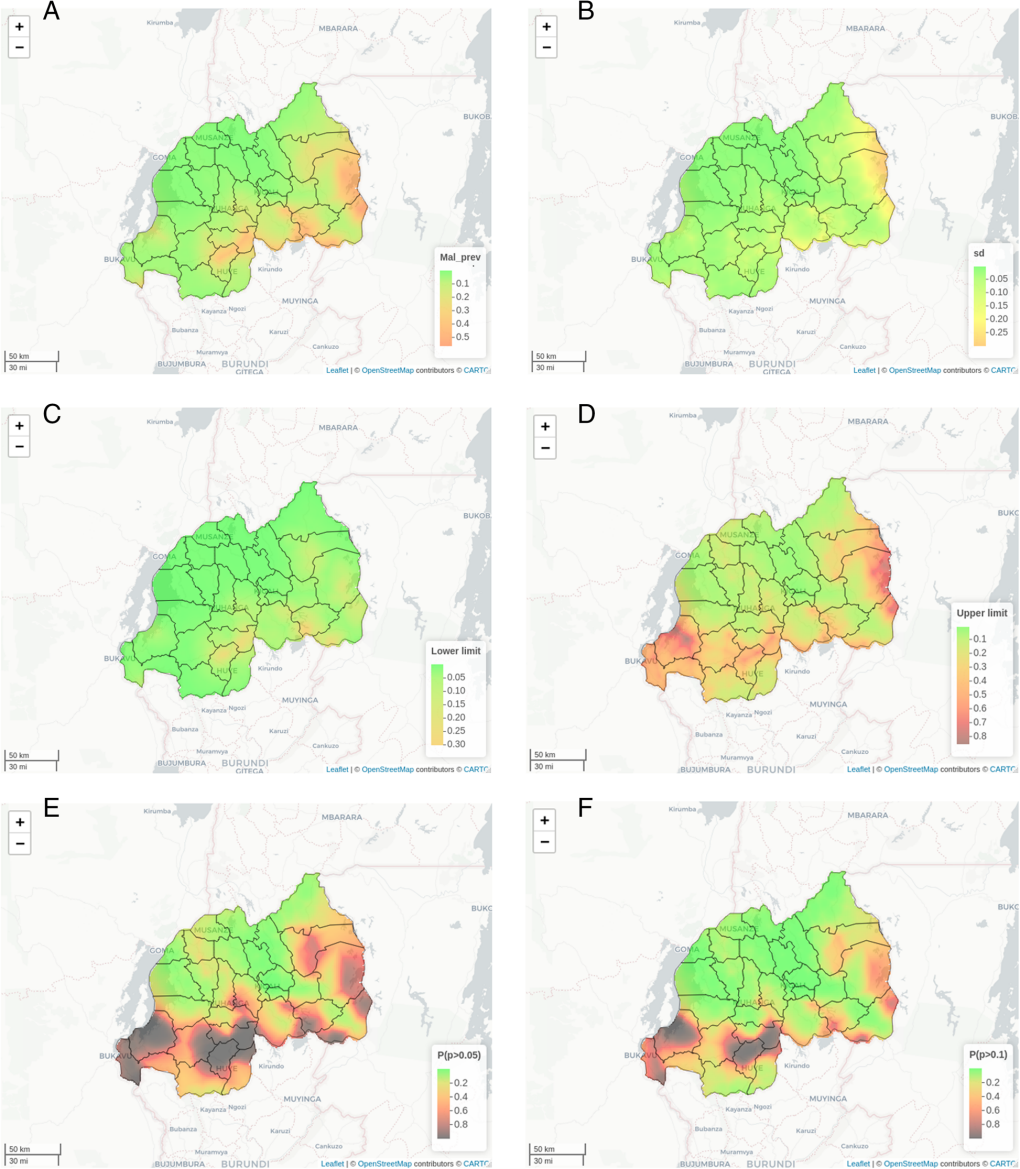
Spatial variation of malaria prevalence among under-five children in Rwanda: Posterior means of spatial effects **A**, Standard deviation **B**, Lower limit **C**, Upper limit **D**, Exceedence probability at 5% **E**, Exceedence probability at 10% **F** (Source: Authors’ creation)

The results of the spatial effects presented in Fig. 3, showing the maps of the posterior mean (A), standard deviation (B), 95% credible intervals (C & D) and those of the exceedance probabilities (E & F). The findings reveal the existence of variation in the prevalence of malaria among under-five children at smaller spatial units in Rwanda. Notably, children from both Eastern and Southern provinces were more likely to suffer from malaria, but those from Northern province, Kigali city, and most parts of the Western province were less likely. The maps of the standard deviation and credible intervals demonstrate higher uncertainties about malaria prevalence in some parts of the country especially around the Eastern and Northern provinces.

To identify the locations that are likely to have an unusual elevation in malaria prevalence at a given threshold, the estimated exceedance probabilities at 5% and 10% thresholds are presented in Fig. 3(E) and (F) respectively. The findings provide evidence of excess under-five malaria prevalence within some of the regions. Locations with exceedance probability closer to 1 (darker) are very likely to experience malaria prevalence exceeding the given threshold while locations whose exceedance probabilities are closer to 0 (green) are unlikely to record malaria prevalence exceeding the thresholds. Districts such as Nyamasheke and Rusizi from Western province; Huye, Nyamagabe, Nyanza and some parts of Nyaruguru and Gisagara from Southern province; Ngoma, Kayonza, Kirehe and parts of Gatsibo from Eastern province are highly likely to have malaria prevalence exceeding 5%. However, when the threshold was raised to 10%, the pattern changed such that locations around Nyamasheke, Rusizi, and Huye, and parts of Gisagara, Kayonza and Kirehe have higher chances of reporting about 10% malaria prevalence.

## Discussion

A geostatistical modeling technique has been adopted to map malaria prevalence in under-five children in Rwanda. The modeling process was based on the SPDE that estimates prevalence at smaller geographical units and identifies the risk factors associated with malaria among under-five children in the country. Furthermore, among the climate conditions, the effects of temperature and rainfall have been evaluated. The study findings have practical relevance as they provide evidence for effective implementation of existing and new intervention programs designed to eradicate malaria in the country.

The linear effects have demonstrated the significant relationship between demographic characteristics of the children and malaria outcomes. Place of residence, mother’s educational level, household wealth index, child’sex, and ownership and use of the treated bed net are found to be significantly associated with the likelihood of contracting malaria. The study findings illustrate that children whose mothers attained at least secondary educational level are less likely to test positive to malaria compared with those whose mothers have no education. Higher educational level among mothers and caregivers has been linked with improved knowledge, perception, and practice concerning strategies for preventing and treating malaria and other diseases [30–32]. There was a significant difference in the likelihood of having malaria between children residing in rural and urban areas. The likelihood is higher among children residing in rural areas than for those residing in urban areas. Studies from other African countries have reported similar differentials in the likelihood contracting malaria by young children based on their type of place of residence [33, 34]. Poor perception and knowledge of malaria and its control measures could be rampart among people living in rural areas. In most rural settings in Rwanda, where more than 83% of the populace live, the majority of parents and guardians are farmers and small scale traders who possess limited access to information on malaria prevention methods [35]. This is aggravated by the lack of adequate health professionals and healthcare systems in rural areas compared with urban centres, increased workload for the available few, and inadequate skills by community health workers in most rural communities.

The study findings also established the significant association between household wealth index and the likelihood of having malaria. Under-five children from the poorest households were having higher chances of suffering from malaria. In Rwanda, the same results have been published, indicating that malaria prevalence in under-five children is more than six times higher among those from the lowest wealth quintile when compared with children from the highest wealth households [15]. A study conducted in Tanzania revealed similar significant association between household wealth index and malaria prevalence among under-five children [36]. Ownership and use of bed nets are also significant risk factors of malaria prevalence among under-five children in Rwanda. Similar findings from Nigeria, Cameroon, and Ghana have shown that children who regularly sleep under bed nets are less likely to test positive for malaria [34, 37, 38]. Like most other African countries attempting to bring malaria under control, Rwanda has adopted, as a key tool in the fight against malaria, the use of insecticide-treated nets. The objective is to attain universal coverage in ownership and use through continuous distribution and mass campaigns. Consequently, there is a regular comparison of access and use indicators, and a focus on behavioral change in order to identify and eliminate any substantial difference across subgroups of the population. Findings on the child’s sex reveal a significant difference in the likelihood of having malaria between male and female children. While a previous study from Rwanda reported similar findings, indicating higher likelihood among male children [11], this was not the case for a study conducted among Kenyan children [39].

The analysis of nonlinear effects of the continuous covariates demonstrates the forms of relationships between child’s age, age of household head, temperature, and rainfall, and malaria among under-five children in Rwanda. The study findings demonstrate that younger children are less likely to test positive, but the risk increases as the child advances in age. This could be attributed to the multiple benefits of breastfeeding, which allows younger children to enjoy the protective ability of breast milk coupled with maternal immunity. These findings are consistent with various studies done elsewhere in Africa [31, 40]. Temperature was found to be positively related with the likelihood of having malaria among under-five children in Rwanda. An increase in temperature reduces the time for the assembly of new generation of mosquitoes and also shortens the incubation period of the parasite in mosquitoes. Using an ecological model with large data on malaria transmission risk from African countries, Modecai et al. [41] demonstrated that the optimal malaria transmission was at temperature of 25^∘^*C* but that transmission decreases dramatically at temperature greater than 28^∘^*C*. A study by Mohammadkhani et al. [42] revealed that with increase in monthly mean temperature, the incidence rate of malaria increased significantly in Iran such that a 1^∘^*C* increase in maximum temperature in a particular month could account for 15% increase in malaria in the same and subsequent month, but that temperatures above 30^∘^*C* and below 16^∘^*C* could harm parasite development. The findings on rainfall demonstrate lower malaria prevalence with increasing volume of rainfall which are consistent with those obtained in other countries including Burundi and Swaziland [43, 44]. However, it has been reported that severe flooding, resulting from extreme rainfall, caused malaria outbreak in highland areas of western Uganda [45]. The amount of rainfall can determine the abundance of mosquitoes and the length of malaria transmission season in different sites such that in lower altitude regions, the rise in rainfall might increase available anopheles sites.

The findings revealed spatial differential in malaria prevalence among under-five children in Rwanda with districts from the Eastern province and some from Southern province including Nyanza and Huye being at high risk. Although various prevention strategies including the use of Long Lasting Insecticidal Treated Nets (LLINs) have been in place since 2006; Indoor Residual Spraying (IRS) since 2007; Integrated Vector Management (IVM) and insecticide resistance mitigation since 2014; and Behavior Change Communication (BCC) implemented mostly targeting high transmission areas to lower morbidity and mortality, malaria cases have increased threefold in the country between 2014 and 2017 [46]. This calls for considerable efforts that targets specific high risks areas as identified in this study. Mapping of exceedance probabilities demonstrates consistent high prevalence of malaria in most districts from Eastern and Southern provinces including some parts of Rusizi and Nyamasheke from the Western province. This demonstrates that malaria prevalence and, indeed, other infectious diseases can vary even among proximate locations. Though malaria prevalence is unlikely to attain 10% in most of the districts, four districts, namely Huye and Nyanza from Southern provinve, and Rusizi and Nyamasheke from Western province have high likelihood of exceeding this threshold. Moreover, some districts including Kayonza and Kirehe from Eastern province; Huye and Nyanza from Southern province, and Nyamasheke and Rusizi from Western province are very likely to have malaria prevalence exceeding 5%. The high risk of malaria in district located in the Eastern and Southern provinces may be explained by irrigation schemes, cross-border movement, and poor housing wall materials that include sprawling human settlements near the marshlands. Notwithstanding that in response to food shortage, Rwanda adopted irrigation-based agricultural practices mostly in the wetlands of the East and South regions, it has been revealed that irrigation practices highly influence malaria by creating vector breeding sites [47]. The spatial variation can also be explained by various factors including spatial differential in human behavior, awareness, and media exposure to malaria information [14].

An important strength of the SPDE model implemented through the R-INLA is the ability of predict the prevalence across all the spatial domain based on sampled observations from specific locations. The model can also be used to handle variables in more complex forms including, for instance, an areal spatial and spatial interactions components, beyond what has been considered in this study. However, as with other studies that rely on data from cross section survey, it is not possible to make causal inference based on the findings. However, the study provides insight on how appropriate intervention programs and resource allocation should be managed in response to high risk areas.

## Conclusion

This study identified that place of residence, child’s age and sex, mother’s educational level, household wealth index, ownership and use of bed nets, and age of household head have direct link with the risks of malaria among under-five children in Rwanda. Moreover, while increase in temperature is associated with rising cases of malaria, the reverse is the case for rainfall. An important finding from the study is the identification of substantial spatial variation in the prevalence of malaria among the young children in the country with different pattern of prevalence, in some cases, among children living within the same districts. The study further identified specific locations where the prevalence of malaria exceeds the threshold of 5% and 10%. Locations around Nyamasheke and Rusizi, Huye, and parts of Gisagara, Kayonza and Kirehe have higher chances of reporting more than 10% malaria prevalence. Considerable efforts to improve the existing control strategies and intervention programs should consider according priority to these areas in fighting malaria in Rwanda. Potential interventions in individuals living in rural areas, lowest wealth quintile, and the area with high risk should be considered when it comes to the formulation of malaria intervention programs. Moreover, the study calls for consideration of climatic and environmental conditions when malaria intervention programs are formulated in Rwanda.

## Data Availability

The data are openly available upon request from The DHS Program (www.dhsprgram.com)

## Abbreviations

US: United Stated
RDHS: Rwanda Demographic and Health Survey
RBC: Rwanda Biomedical Center
SPDE: Stochastic Partial Differential Equation
MoH: Ministry of Health
INLA: Integrated Nested Laplace Approximation
NISR: National Institute of Statistics of Rwanda
EAs: Enumeration Areas
RPHC: Rwanda Population and Housing Census
RDT: Rapid Diagnostic Test
GPS: Global Positioning System
LLINs: Long Lasting Insecticidal treated Nets
IRS: Indoor Residual Spraying
IVM: Integrated Vector Management
BCC: Behavior Change Communication
GRF: Gaussian Random Field
GMRF: Gaussian Markov Random Field
